# Supplementary material for: Rice Fields as Important Habitats for Three Anuran Species—Significance and Implications for Conservation
Source: Animals (Basel). 2023 Dec 27;14(1):106. doi: 10.3390/ani14010106 (PMC10778459; doi:10.3390/ani14010106)
Supplement: Supplementary file 1 [file animals-14-00106-s001.zip › S2 Water_parameter_values.pdf]

Table S1. Values of the indicators measured in the water sample analyses for 2022 (C – control pond, R1- rice field 1, R2 – rice field 2). Data are presented as Mean ± SD, statistically significant differences between sites are in bold and italic.

	Indicator	Units	June			September	
			C	R1	R2	R1	R2
1	Enterococci count	n/100ml	***18 ± 400***	***1380 ± 158***	***280 ± 68***	180 ± 60	190 ± 80
2	Clostridium perfringens count	n/100ml	0	0	***20 ± 10***	0	4 ± 1
3	Organic carbon	mg/l	***16.5 ± 2.8***	***7.56 ± 1.2***	***2.96 ± 0.41***	4.8 ± 1	5.07 ± 1.1
4	Nitrates	mg/l	0	0	0.96 ± 0.184	0	0
5	Chlorides	mg/l	**27 ± 3.2**	**3.9 ± 0.5**	**9.76 ± 1.12**	16 ± 2	11 ± 1
6	Fluorides	mg/l	***0.81 ± 0.08***	***0.22 ± 0.02***	***0.16 ± 0.018***	0.24 ± 0.03	0.39 ± 0.04
7	Nitrites	mg/l	0	0	0.016 ± 0.004	0	0
8	Phosphates	mg/l	1 ± 0.3	0	0	0	0
9	Sulfates	mg/l	***99 ± 9***	***64 ± 6***	***54 ± 4.37***	***79 ± 4***	***3.6 ± 0.4***
10	Bromates	µg/l	0	0	0	0	0
11	Hardness	meq/l	***4.8 ± 0.4***	2.4 ± 0.2	2.17 ± 0.23	3.1 ± 0.3	3.3 ± 0.3
12	Cyanides	mg/l	0	0	0	0	0
13	Calcium	mg/l	***77 ± 7***	33 ± 3	35 ± 3	41 ± 4	45 ± 5
14	Magnesium	mg/l	***18 ± 1.6***	***9.4 ± 0.9***	***6.3 ± 0.6***	11 ± 0.9	11 ± 0.9
15	Sodium	mg/l	***65 ± 5***	12 ± 1	9.6 ± 0.8	14 ± 1	10 ± 0.8
16	Boron	mg/l	0.04 ± 0.004	0.03 ± 0.003	0.01 ± 0.002	0.012 ± 0.002	0.021 ± 0.002
17	Cadmium	mg/l	0	0	0	0	0
18	Copper	mg/l	0.023 ± 0.003	0.004 ± 0.0004	0.011 ± 0.001	0.003 ± 0.0003	0
19	Nickel	mg/l	0.002 ± 0.0002	0.003 ± 0.0003	0.001 ± 0.0003	0	0
20	Lead	mg/l	0	0	0	0	0
21	Chromium	mg/l	0	0	0	0	0
22	Zinc	mg/l	0	0	0	0	0
23	Manganese	mg/l	***15.231 ± 1.95***	***0.334*** ± 0.039	***0.042 ± 0.004***	***0.013 ± 0.001***	***0.452 ± 0.053***
24	Antimony	mg/l	0.001 ± 0.0001	0.006 ± 0.0006	0.001 ± 0.0005	0	0
25	Arsenic	mg/l	***0.019 ± 0.002***	***0.003*** **± 0.0003**	***0.002 ± 0.0006***	0.003 ± 0.0003	0.003 ± 0.0003

26	Selenium	mg/l	0	0	0	0	0
27	Mercury	mg/l	0	0	0	0	0
28	Natural uranium	mg/l	0	0	0	0	0
29	Trichloroethene	µg/l	0	0	0	0	0
30	Tetrachloroethene	µg/l	0	0	0	0	0
31	Chloroform	µg/l	0	0	0	0	0
32	Bromoform	µg/l	0	0	0	0	0
33	Bromodichloromethane	µg/l	0	0	0	0	0
34	Dibromochloromethane	µg/l	0	0	0	0	0
35	Trihalomethanes	µg/l	0	0	0	0	0
36	1,2-dichloroethane	µg/l	0	0	0	0	0
37	Benzene	µg/l	0	0	0	0	0
38	p,p-DDE	µg/l	0	0	0	0	0
39	p,p-DDT	µg/l	0	0	0	0	0
40	p,p-DDD	µg/l	0	0	0	0	0
41	Methoxychlor	µg/l	0	0	0	0	0
42	Heptachlor	µg/l	0	0	0	0	0
43	Heptachlor epoxide	µg/l	0	0	0	0	0
44	Aldrin	µg/l	0	0	0	0	0
45	Endrin	µg/l	0	0	0	0	0
46	Endrin aldehyde	µg/l	0	0	0	0	0
47	Dieldrin	µg/l	0	0	0	0	0
48	Endosulfan I	µg/l	0	0	0	0	0
49	Endosulfan II	µg/l	0	0	0	0	0
50	Endosulfan sulfate	µg/l	0	0	0	0	0
51	alpha-HCH	µg/l	0	0	0	0	0
52	beta-HCH	µg/l	0	0	0	0	0
53	gamma-HCH	µg/l	0	0	0	0	0
54	delta-HCH	µg/l	0	0	0	0	0
55	Organochlorine pesticides	µg/l	0	0	0	0	0

56	Benzo[a]pyrene	µg/l	0	0	0	0	0
57	Benzo[b]fluoranthene	µg/l	0	0	0	0	0
58	Benzo[k]fluoranthene	µg/l	0	0	0	0	0
59	Benzo[ghi]perylene	µg/l	0	0	0	0	0
60	Indeno[1,2,3-cd]pyrene	µg/l	0	0	0	0	0
61	Aromatic hydrocarbons	µg/l	0	0	0	0	0
62	Epichlorohydrin	µg/l	0	0	0	0	0

Table S2. Values of the indicators measured in the water sample analyses for 2023 (C – control pond, R1- rice field 1, R2 – rice field 2). Data are presented as Mean ± SD, statistically significant differences between sites are in bold and italic.

	**Indicator**	**Units**	**June**			**September**	
			**C**	**R1**	**R2**	**R1**	**R2**
1	Enterococci count	n/100ml	***20 ± 9***	***210 ± 90***	***755 ± 320***	180 ± 70	290 ± 120
2	Clostridium perfringens count	n/100ml	***0***	***30 ± 19***	***170 ± 110***	0	0
3	Organic carbon	mg/l	15.6 ± 2.5	15.4 ± 2.5	13.2 ± 2.1	5.05 ± 0.75	4.74 ± 0.7
4	Nitrates	mg/l	0	0	1 ± 0.1	0	0.97 ± 0.1
5	Chlorides	mg/l	**29 ± 3**	9.9 ± 0.9	13 ± 1	12 ± 1	13 ± 1
6	Fluorides	mg/l	***0.95 ± 0.09***	***0.38 ± 0.04***	***0.25 ± 0.02***	0.29 ± 0.03	0.31 ± 0.03
7	Nitrites	mg/l	0	0.041 ± 0.003	0.069 ± 0.005	0	0.019 ± 0.002
8	Phosphates	mg/l	0	0	0	0	0.11 ± 0.01
9	Sulfates	mg/l	***91 ± 5***	***48 ± 3***	***220 ± 12***	59 ± 3	44 ± 2
10	Bromates	µg/l	0	0	0	0	0
11	Hardness	meq/l	5.1 ± 0.5	***1.7 ± 0.2***	4.7 ± 0.4	2.3 ± 0.2	3 ± 0.3
12	Cyanides	mg/l	0	0	0	0	0
13	Calcium	mg/l	70 ± 7	***25 ± 3***	72 ± 7	32 ± 3	43 ± 4
14	Magnesium	mg/l	19 ± 2	***5.9 ± 0.5***	13 ± 1	8 ± 0.7	9.8 ± 0.8
15	Sodium	mg/l	***71 ± 6***	***8 ± 0.7***	***15 ± 1***	13 ± 1	13 ± 1
16	Boron	mg/l	***0.032 ± 0.006***	***0.015 ± 0.003***	***0.042 ± 0.007***	0.013 ± 0.002	0.022 ± 0.004
17	Cadmium	mg/l	0	0	0	0	0

18	Copper	mg/l	0.017 ± 0.002	***0.005 ± 0.0004***	0.01 ± 0.0009	0.003 ± 0.0003	0.004 ± 0.0003
19	Nickel	mg/l	0.002 ± 0.0002	0.002 ± 0.0002	0.005 ± 0.0005	0	0
20	Lead	mg/l	0	0	0	0	0
21	Chromium	mg/l	0	0.001 ± 0.0002	0	0	0
22	Zinc	mg/l	0	0	0	0	0
23	Manganese	mg/l	***13.122 ± 2.5***	***0.006 ± 0.0008***	***0.028 ± 0.005***	***0.033 ± 0.006***	***0.006 ± 0.0007***
24	Antimony	mg/l	0.002 ± 0.0002	0.003 ± 0.0003	0.002 ± 0.0002	0	0
25	Arsenic	mg/l	0.014 ± 0.002	0.003 ± 0.0004	0.002 ± 0.0003	0.003 ± 0.0004	0.001 ± 0.0002
26	Selenium	mg/l	0	0	0	0	0
27	Mercury	mg/l	0	0	0	0	0
28	Natural uranium	mg/l	0	0.002 ± 0.0002	0	0	0
29	Trichloroethene	µg/l	0	0	0	0	0
30	Tetrachloroethene	µg/l	0	0	0	0	0
31	Chloroform	µg/l	0	0	0	0	0
32	Bromoform	µg/l	0	0	0	0	0
33	Bromodichloromethane	µg/l	0	0	0	0	0
34	Dibromochloromethane	µg/l	0	0	0	0	0
35	Trihalomethanes	µg/l	0	0	0	0	0
36	1,2-dichloroethane	µg/l	0	0	0	0	0
37	Benzene	µg/l	0	0	0	0	0
38	p,p-DDE	µg/l	0	0	0	0	0
39	p,p-DDT	µg/l	0	0	0	0	0
40	p,p-DDD	µg/l	0	0	0	0	0
41	Methoxychlor	µg/l	0	0	0	0	0
42	Heptachlor	µg/l	0	0	0	0	0
43	Heptachlor epoxide	µg/l	0	0	0	0	0
44	Aldrin	µg/l	0	0	0	0	0
45	Endrin	µg/l	0	0	0	0	0
46	Endrin aldehyde	µg/l	0	0	0	0	0
47	Dieldrin	µg/l	0	0	0	0	0

48	Endosulfan I	μg/l	0	0	0	0	0
49	Endosulfan II	μg/l	0	0	0	0	0
50	Endosulfan sulfate	μg/l	0	0	0	0	0
51	alpha-HCH	μg/l	0	0	0	0	0
52	beta-HCH	μg/l	0	0	0	0	0
53	gamma-HCH	μg/l	0	0	0	0	0
54	delta-HCH	μg/l	0	0	0	0	0
55	Organochlorine pesticides	μg/l	0	0	0	0	0
56	Benzo[a]pyrene	μg/l	0	0	0	0	0
57	Benzo[b]fluoranthene	μg/l	0	0	0	0	0
58	Benzo[k]fluoranthene	μg/l	0	0	0	0	0
59	Benzo[ghi]perylene	μg/l	0	0	0	0	0
60	Indeno[1,2,3-cd]pyrene	μg/l	0	0	0	0	0
61	Aromatic hydrocarbons	μg/l	0	0	0	0	0
62	Epichlorohydrin	μg/l	0	0	0	0	0

Table S3. Standard codes and short description of the testing methods used for the measurement of the indicators in the water samples.

№	Standard	Short description
1	ISO 7899-2:2003	Detection and enumeration of intestinal enterococci - membrane filtration method.
2	ISO 14189:2013(E)	Enumeration of *Clostridium perfringens* - membrane filtration method.
3	EN 1484:2004	Determination of total organic carbon and dissolved organic carbon by means of combustion oxidation.
4-7, 9	ISO 10304-1:2009	Determination of dissolved anions by liquid chromatography of ions.
8	SO 6878:2005	Determination of phosphorus - ammonium molybdate spectrometric method.
10	№PW-36/01.11.2012	Determination of bromates in water with an ion chromatograph.
11, 13-15	ISO 14911:2002	Determination of dissolved cations using ion chromatography.
12	ISO 6703-1:2002	Determination of total cyanide by photometric and titrimetric methods.
16-28	ISO 17294-2:2016	Application of inductively coupled plasma mass spectrometry - determination of selected elements including uranium isotopes.

29-37	ISO 15680:2004	Gas-chromatographic determination of monocyclic aromatic hydrocarbons, naphthalene and chlorinated compounds using purge-and-trap and thermal desorption.
38-55	№PW-29/01.09.2010	Determination of highly volatile halogenated hydrocarbons in water by means of gas chromatography with mass spectrometric detection.
56-61	ISO28540:2011(E)	Determination of polycyclic aromatic hydrocarbons in water using gas chromatography with mass spectrometric detection.
62	EPA 8260C	Determination of volatile organic compounds by means of gas chromatography with mass spectrometric detection.
